# PROTOCOL: Medical‐financial partnerships for improving financial and health outcomes for lower‐income Americans: A systematic review

**DOI:** 10.1002/cl2.1364

**Published:** 2023-10-09

**Authors:** Julie Birkenmaier, Brandy Maynard, Hannah Shanks, Harly Blumhagen

**Affiliations:** ^1^ Saint Louis University School of Social Work St Louis Missouri USA

## Abstract

This is the protocol for a Campbell systematic review. The primary objectives of this review is to answer the following research questions using formal research studies: What is the extent and quality of MFP intervention research? What are the effects on financial outcomes of financial services embedded within healthcare settings? What are the effects on health‐related outcomes of financial services embedded within healthcare settings?

## 1 BACKGROUND

Poverty is a widespread problem in the United States. About 12% of Americans, or 38 million people, have household incomes below the US poverty line (US Census Bureau, 2022). The health problems associated with poverty are so significant that poverty is considered one of the social determinants of health, which refers to a broad range of social and environmental conditions that affect health and well‐being (Francis et al., 2018). Living in poverty has major effects on people's physical health because poverty affects a person's access to safe housing, healthy foods, employment and educational opportunities, healthcare services, clean air and water, and a safe neighborhood environment. Living in poverty affects both adults and children: growing up in poverty without access to these things negatively affects children's health and well‐being (US Department of Health and Human Services, n.d.). Poverty is a special concern for minoritized populations, as poverty disproportionately impacts racial and ethnic minorities (Board of Governors of the Federal Reserve, 2021. The effect of finances on health has been demonstrated at the policy level as well: Bradley et al. (2016) demonstrated that states with more social services that affect income and finances had significantly better health outcomes.

Both those living below and above the federal poverty level, which is $30,000 annually for a family of four in 2023, experience financial stress and strain. Even those households that are able to meet daily and monthly expenses experience financial stress when an unexpected expense occurs. Most US households have less short‐term savings than the amount needed to cover expenses for 3 months in the absence of income, as recommended by financial planners (FINRA Investor Education Foundation, 2019). Only 68% of adults in 2021 could cover a hypothetical expense of $400 using cash, savings, or a credit card paid off at the next statement (Board of Governors of the Federal Reserve, 2022). Households with financial need, but without savings, often take on debt, which is unmanageable for the 28% of the population who have unmanageable debt that has resulted in debt collections documented on their credit report (Carther et al., 2022). The lack of savings is also critical for older adults; half of all adults nearing retirement have no personal retirement funds (King, 2022).

Financial stress and strain negatively affects health and well‐being through several pathways. First, chronic stress leads to the acceleration of normal aging through shortening of telomeres and increases in allostatic load. These processes, called “weathering,” lead to earlier onset of unfavorable physical health conditions (Forde et al., 2019). Second, financial stress disrupts medical care due to foregone care as a result of families neglecting their health because they cannot afford to seek preventative or early care, or experience missed clinic visits due to cost or other poverty‐related access challenges. Financial stress can force families to focus on basic needs that may be more urgent than preventive medical care visits, which often results in symptoms worsening and thus requiring more expensive or prolonged care (Schickedanz et al., 2023). Financial stress can be compounded for people of color and those who live in communities of color caused by their encounters with racial and economic discrimination (Geronimus, 2023). Children in low‐income households whose parents are under financial stress may suffer harm to their cognitive and social/emotional development due to harsh, inconsistent and detached parenting (National Academies of Sciences, Engineering, and Medicine, 2019).

Various interventions exist to try to alleviate poverty and financial stress for financially vulnerable populations. For example, financial counseling, mentoring, and coaching are interventions delivered in a variety of formats and settings, including schools, nonprofit organizations, the military, and employers. Other interventions combine financial incentives and financial education or information, such as Individual Development Accounts and Child Development (or Savings) Accounts (Birkenmaier et al., 2022). Another type of intervention is focused on tax preparation and filing. One intervention provides free tax preparation services for low‐income families, while another combines tax preparation with encouragement and behavioral nudges to save money or purchase safe investments, such as Certificates of Deposit (CDs) (Birkenmaier et al., 2023).

A recent discussion occurring in the public and academic arenas is focused on the potential role of healthcare settings in addressing financial stress and poverty, along with the other determinants of health, to improve health outcomes. Both primary and tertiary healthcare settings that target and/or serve primarily lower‐income and communities of color are well‐positioned to provide services to lower financial stress (South et al., 2022). Primary healthcare settings provide low‐ or no cost preventive and primary healthcare, while tertiary healthcare settings provide a higher level of care within a hospital. Because patients can access the services of primary and tertiary healthcare providers directly without a referral, they are often the first point of contact in the healthcare system. Since primary healthcare addresses the comprehensive and interrelated aspects of physical, mental, and social health, primary healthcare providers are in settings to learn more about the home environment and stressors from patients. Tertiary healthcare settings with hospital clinics and wellness centers can also incorporate financial services to reduce financial stress (Bell et al., 2020).

Parallel practice discussions are focused at improving health outcomes. Primary healthcare settings are seeking ways to increase the rate at which people fulfill their appointments. Primary healthcare clinics that serve low‐income families have no‐show rates for appointments of 25%–50%, which is much higher than rates for higher‐income families (Samuels et al., 2015). Few interventions to date have shown a substantial improvement in increasing preventative health visits or lower reliance on emergency room visits or improving care visit show rates (Macharia, 1992). Medical financial partnerships (MFPs) are an intervention targeted at both financial stress and health outcome goals.

### 1.1 The intervention

In recent years, MFPs have been piloted to formally address financial stress and poverty within healthcare settings that serve lower‐income and Medicaid‐insured patients (Liu et al., 2021), and also encourage keeping healthcare appointments. MFPs are collaborations between financial service organizations and health clinics, hospitals, or health systems to implement services that improve both the finances and health of a community, and that directly impact financial stability as part of the healthcare model (Bell et al., 2020; Hole et al., 2017). They build an intentional connection between health care delivery and financial services, and may include a limited or broader range of services (Marcil et al., 2021). The goal of service provided is to build patient financial stability. Services may include free tax preparation, enrollment in savings accounts, employment assistance and workforce development, financial coaching, and/or assistance with applying for benefit programs. MFPs serve a range of clients to include hospital and clinic patients, employees, and community members. MFPs provide financial services onsite and in the community through formal partnerships (Marcil et al., 2021).

MFPs have been developed in response to several main drivers. First, there is a strong argument that financial stress falls within the concept of primary health care. Professional and government entities, including the Academy of Pediatrics and the American Academy of Family Physicians, call for physicians to address the social determinants of health (American Academy of Family Physicians, 2021; Gitterman et al., 2016). In addition, both the National Association of Community Health Centers and the Centers for Medicare and Medicaid Services have produced screening tools for social determinants of health (Center for Medicare and Medicaid Services, n.d.); National Association of Community Health Centers, Inc. & Association of Asian Pacific Community Health Organizations [NACHC & AAPCHO], 2022). Scholars have also highlighted recent screening tools for the social determinants of health, including poverty (Gruss et al., 2021; LaForge et al., 2018; O'Gurek & Henke, 2018). Second, nonfinancial social resources have long been integrated into primary healthcare settings, such as programs that address food security (Adams et al., 2017; Palakshappa et al., 2017), and provide behavioral health services and legal services (Beck et al., 2021). Some healthcare settings have incorporated Medical‐Legal Partnerships (MLP), which embed legal services addressing health‐harming legal needs, including legal barriers to financial wellness, such as benefit or disability appeals, workplace discrimination, and family medical leave (Marcil et al., 2021). Thirdly, qualitative studies have demonstrated interest in the integration. Low‐income patients of health‐clinics have expressed interest in healthcare setting‐based financial services, such as financial coaching and free tax preparation clinics (Liu et al., 2021). Embedding financial services within health clinics could lead to improved financial outcomes, such as reduced unmanageable debt and higher credit scores, as well as improved health outcomes through reduced financial stress, greater healthcare visit adherence, and higher preventive health care service receipt (Dalembert et al., 2021; Schickedanz et al., 2023).

### 1.2 How the intervention might work

As mentioned, MFP interventions can occur on‐site and off‐site of healthcare settings through partnerships. To differentiate MFP interventions from traditional social work services in healthcare settings, this review will focus on on‐site interventions as a unique intervention, as well as financial services delivered via tele‐consultation with a financial service provider who is providing services within a service delivery model of a healthcare provider. Social workers in healthcare settings often provide referrals to off‐site community resources, such as financial counseling and coaching and emergency nutrition supplements (Browne, 2019). In contrast, MFPs that use colocation differ from traditional social services, and provide unique features. First, colocation provides a convenient way for patients to access services, either in combination with their healthcare services or independently. Second, colocated services are considered part of the healthcare model (Marcil et al., 2021). On‐site MFP interventions can involve varied patient financial services. Services provided by the programs include financial coaching, tax preparation services, credit counseling, debt consolidation and forgiveness, and employment assistance (Bell et al., 2020). These services are offered to patients within the physical space of healthcare settings, such as clinics, offices, and hospitals (Dalembert et al., 2021; San Francisco General Hospital, n.d.), or via tele‐consultation.

To create these colocated services, healthcare settings must create community partnerships. Healthcare settings can use a model for developing and sustaining effective community partnerships created by the American Academy of Pediatrics. In this model, healthcare providers can identify partners, align their needs, ensure patient benefit, sustain patient bonds, and manage staff time for the most effective service delivery (American Academy of Pediatrics, 2023). In the day‐to‐day provision of services, medical providers, as well as front desk staff and greeters, provide information about the financial services offered on‐site or via tele‐consultation, and encourage patients to consider using them.

The two most prominent services are financial coaching and tax preparation services (Dalembert et al., 2021; Marcil et al., 2021). Financial coaching has grown in recent years as a standardized, evidence‐based, and strengths‐based approach to assist low‐income families in achieving their potential for financial well‐being (Hall et al., 2022; Modestino et al., 2019). The method entails client‐driven goal setting, along with other needed services, such as financial education, and assistance in improving budgeting, savings, and credit. Financial coaches assist clients to set their goals, then offer one‐on‐one appointments that include motivational interviewing, financial planning, and connection with public benefits (Collins et al., 2021). Financial coaching has been shown to increase financial knowledge and confidence, reduce financial stress, increase income and savings, reduce debt, and improve credit (Collins et al., 2021; Silva et al., 2022).

In the MFP model, financial coaches may communicate with participants between clinic visits, focused on financial stressors and patient‐centered goal progress. The focus is often on building supportive, trusting, nonjudgmental relationships to support patient financial capabilities and strengths to reduce barriers to visit adherence. The main focus is to build trust in the health care team and their financial coaches, and generate new motivation for making both their healthcare and financial services appointments by aligning services provided with their self‐identified financial goals. This trust‐building hopefully has positive effects on their healthcare visit adherence (Schickedanz et al., 2023).

Free tax preparation is another common financial intervention provided within the MFP model (Bell et al., 2020). Due primarily to two key refundable tax credits (i.e., the Earned Income Tax Credit and the Child Tax Credit), filing federal income taxes can provide the largest one‐time influx of cash that low‐income working households receive annually. However, annually, approximately 20% of eligible people do not receive funds to which they are entitled (Internal Revenue Service, 2022), and Black and Latinx children are disproportionately likely to be in families that do not file taxes and receive these benefits (Bruenig & Williams, 2021). Tax preparation through the federal Volunteer Income Tax Assistance (VITA) program or Tax Counseling for the Elderly (TCE) programs offers free tax preparation to people who earn $60,000 or less annually, people with disabilities, and taxpayers with limited English proficiency (US Department of Housing and Urban Development [U.S. HUD], n.d.). These services could save as much as the $1.75 billion earned by the for‐profit tax industry annually (Weinstein & Patten, 2016).

Other services provided, in decreasing order of the number of MFPs that provide them, are employment assistance, diaper distribution, food insecurity assistance, enrollment in Child Development Accounts, housing assistance, enrollment in matched savings accounts programs, benefits enrollment, FAFSA form assistance, pre‐k enrollment assistance and medical bill arbitration (Marcil et al., 2021).

### 1.3 Theory of change

MFPs help families reduce financial stress and poverty, and grow assets in a number of ways. Similar to financial services provided outside of a healthcare setting, these services provide resources, knowledge, and skills to reduce expenses, increase income, decrease debt, increase credit score, and/or increase savings. In addition, the colocation of services may increase the perceived value and relevance of the visit to the healthcare provider, and/or reduce the poverty‐related barriers to visit adherence, such as transportation problems and inability to afford time off work to receive services individually. Receiving financial services in conjunction with a trusted clinical setting may deepen patients’ relationships and trust with the setting (Marcil et al., 2018; Markowitz et al., 2022). Providing empowering, nonjudgmental, expert, and trustworthy financial services in a convenient location may help them to set and achieve financial goals while also fulfilling their healthcare visit needs (Alexander et al., 2022).

In pediatric settings and for parents of pediatric patients, it may also reduce indirect barriers such as competing demands on parent's time and attention from meeting basic needs, such as housing and food (Quinn et al., 2018). Healthcare is one of the few sectors that reach the majority of families with children (86.5% in 2018), so the intervention is in a setting reaching a large portion of the population (Centers for Disease Control and Prevention, 2020).

### 1.4 Why it is important to do the review

This review has relevance to policy related both to health and finances. First, health policy is increasingly recognizing the social determinants of health, such as finances, as important influences on health (World Health Organization, 2023). Addressing financial goals and needs of patients, which could lead to improved health outcomes, may impact policy through reduced healthcare costs that would otherwise result from forgone preventative healthcare, such as vaccinations and health screenings. Health policy, especially related to primary healthcare settings, could be influenced by study outcomes. Second, there is currently little evidence supporting the idea that financial services, such as financial coaching and free‐tax preparation clinics, could magnify their effectiveness by becoming embedded in frequented and trusted settings, such as health clinics (Alexander et al., 2022). Evidence related to the effectiveness of embedding financial services could shape policy related to these services. For example, evidence of the effectiveness of MFPs could encourage policymakers to incorporate them more fully into health systems and create reimbursement avenues for financial services.

Despite the attention that the MFP intervention has received recently the research appears largely scattered, and to date, there is no systematic review of the MFP interventions related to their financial and health outcomes. One scoping review (Parry et al., 2021) studied all types of financial interventions within healthcare systems to address poverty in high‐income countries. However, systematic review methods were not followed. Bell et al. (2020) conducted a systematic review of the types of MFPs models and characterized different MFP organizational models. However, no systematic reviews, to date, have examined their financial and/or health outcomes. The research in this area is also nascent, with the first academic articles on the topic appearing in 2017. In our initial search for studies, we located several quantitative studies (Marcil et al., 2018, 2021; Markowitz et al., 2022; Schickedanz et al., 2023) and several qualitative studies (Bennett et al., 2021; Jaganath et al., 2018; Liu et al., 2021; Quinn et al., 2018), however, the evidence is scattered across different disciplines and our search for primary studies was not extensive to this point. We expect to find additional studies as we improve and extend our search, particularly to include gray literature. Because the research is scattered across disciplines, and these interventions are relatively new, it is important to conduct a systematic review to examine the types of interventions being implemented, the outcomes being measured, and the quality and strength of the evidence to most effectively and efficiently inform policy and practice of this multidisciplinary intervention intended to impact health, financial outcomes and well‐being.

We intend to cover knowledge gaps in several ways. First, while individual studies on some of these interventions offered within MFPs have found positive effects (e.g., financial coaching, Theodos et al., 2015; e.g., tax‐time saving intervention, Roll et al., 2020), recent systematic reviews on other interventions offered within MFPs, such as matched savings accounts (Birkenmaier, Kim, et al., 2022), Child Development Accounts (Birkenmaier et al., 2021), tax‐time saving interventions (Birkenmaier et al., 2023), and on financial services interventions in general, which included financial coaching (Birkenmaier, Maynard, et al., 2022) have found mostly mixed or relatively weak effects on savings amounts or were inconclusive. Other reviews, including systematic reviews, have concluded that financial education, counseling, and coaching has no or small effects on later behavior, including short‐term saving (Birkenmaier, Maynard, et al., 2022; Fernandes et al., 2014; Kaiser & Menkhoff, 2020; Miller et al., 2015). Therefore, additional systematic reviews of financial services interventions are needed.

## 2 OBJECTIVES

The primary objectives of this review is to answer the following research questions using formal research studies:

1. What is the extent and quality of MFP intervention research?
2. What are the effects on financial outcomes of financial services embedded within healthcare settings?
3. What are the effects on health‐related outcomes of financial services embedded within healthcare settings?

## 3 METHODOLOGY

### 3.1 Inclusion criteria

#### 3.1.1 Other inclusion criteria

We will include studies that are conducted any time (no time exclusion). We will search for studies starting in 2017, as, to our knowledge, studies on this intervention started to be produced in 2017. However, we will not exclude studies published earlier.

We will include studies that provide any financial services on site, whether one type (i.e., targeted) or many types (i.e., full‐scope) (Bell et al., 2020).

We will include studies irrespective of whether they are published in journals/books or available as working papers, reports, or other.

We will include only studies with interventions delivered within the United States healthcare system, given its unique structure among healthcare systems in developed countries.

We will include only studies published in English, as we are focused on interventions delivered within the United States.

#### 3.1.2 Types of studies

Study designs to be included are:

To answer the three research questions, we will include studies that meet the following criteria:

1. Randomized controlled trials (RCTs). This type of research study specifically uses a random assignment of participants to condition, and evaluates the effect by comparing the outcome between the treatment and control groups.
2. Quasi‐experimental designs (QEDs). When the assignment to groups is not random, studies must use a design that compares a group of participants who receive the intervention and another group that does not receive the intervention. The comparison group could include those on a waitlist, attention control, or standard care. Comparison or control groups can receive their needed healthcare, but no financial services within the healthcare setting. It is possible that comparison/control group members receive financial services outside of the healthcare setting.

### 3.2 Type of participants

All populations (e.g., children and adults of any age) will be included. All US healthcare clinical settings where primary, secondary, or tertiary healthcare services are provided are eligible to be included. We will exclude studies where the participants are not using a US‐based healthcare setting because of the unique nature of the US healthcare system among other developed countries. We will also exclude MFP studies wherein the financial services were delivered off‐site and those delivered via tele‐consultation outside of a formal partnership with a healthcare organization.

### 3.3 Type of interventions

The eligible intervention will be the provision of financial services within a US clinical healthcare setting. The financial services could be financial education, counseling or coaching, credit counseling, or the provision of services that assist patients to access financial products or services, such as free tax preparation services, matched savings accounts or special child savings accounts to be used for education purposes. The intervention could also include services to increase income, such as screening for public benefits and assistance with the application process, as well as employment services (e.g., assistance with resume writing and job interviewing skills). Financial services could be provided by any group, organization, or agency, whether nonprofit or for‐profit, whose primary role is to provide such services. The services are provided to the patients who use the healthcare setting for any duration of services.

#### 3.3.1 Excluded studies

Similar interventions that are not eligible are:

1. Interventions that provide financial assistance to patients for the sole purpose of affording their healthcare, including medications. These interventions, such as cash assistance to purchase medications or afford copays, or programs that discount medications or the cost of medical services, are solely focused on delivering the needed healthcare services, and not on improving the patient's overall financial situation and reducing financial stress.
2. Interventions that solely involve screening for financial‐related topics while onsite in a healthcare delivery setting.
3. Interventions that involve a partnership between a healthcare provider, insurer or company and a financial services provider that are offered off‐site of a healthcare facility.
4. Interventions that solely provide referrals to financial services or financial assistance (see e.g., McConnell et al., 2020). Examples of these interventions are called “social prescribing”, “clinical‐community linkages” and “social referrals” (Parry et al., 2021). Referring patients to other services is a relatively weak intervention that involves providing information about a service delivered outside of the structure of the healthcare setting. Instead, the focus of this study is to examine the effectiveness of services brought within the structure of a healthcare setting, and focused on longer‐term financial well‐being.
5. Interventions that are solely characterized as “medical‐legal partnerships”. These interventions aim at issues amenable to legal remedies, most often with housing and income issues. In these interventions, legal services are provided to help clients obtain access to external food and income supports, claim public benefits and prevent shut offs of utilities (Klein et al., 2013).
6. Interventions that occur outside of the United States. The US healthcare system is unique among high‐income countries in not providing universal healthcare, and intervention effectiveness may be unique because of the healthcare system.

### 3.4 Type of outcome measures

*To be included in this review, studies must measure and report a financial outcome as a primary outcome. Studies may also report a health care outcome*.

*Specifically*

1. All financial outcomes will be included. These outcomes can include financial knowledge and attitude, tax filing status, tax refund receipt and amount, debt, savings, and credit scores.
2. All reported health outcomes and perceptions about health will be included. These outcomes can include missed visits, immunization schedule adherence, and other health indicators.

We are defining these outcomes very broadly and inclusively since there are a wide range of outcomes that are being measured in the studies we have thus far identified in our initial search to test search terms and gauge the number of expected studies. We want to be able to capture all outcomes related to health and financial outcomes that are being measured.

*If studies include a financial outcome as a primary outcomes listed above and meet other inclusion criteria, we will also capture data related to the following secondary outcomes when reported:*

1. Knowledge about tax filing and tax credits (e.g., EITC, Child Tax Credit) (Marcil et al., 2018).
2. Outcome related to healthcare usage (e.g., feel more connected to healthcare providers) (Marcil et al., 2018).
3. Outcomes related to child patients of participating parents. These outcomes can include education goals for children whose parents participate in a child special savings account program.
4. Outcomes related to employment services. These outcomes can include outcomes related to resume assistance, job search assistance, employment application and enrollment assistance, and income from paid employment.
5. Outcomes related to income security unrelated to employment, such as accessing public benefits.
6. Outcomes related to microfinance and emergency finance.
7. Adverse or unintended outcomes reported in primary studies.

### 3.5 Duration of follow‐up

We will include outcomes measured at post‐intervention and all follow‐up time points.

### 3.6 Search strategy

We will attempt to identify and retrieve both published and unpublished studies through a comprehensive search that includes multiple electronic databases, gray literature sources, reference lists of reviews and relevant studies and inquiry of experts, trial registries and relevant websites (Kugley et al., 2017). The search strategy and results of the search will be documented in sufficient detail to produce a flow chart.

Electronic databases (Platform): ABI/INFORM (ProQuest); Academic Search Complete (EBSCOhost), Business Source Premier (EBSCOhost); CINAHL Plus with Full Text, ProQuest Dissertations & Theses Global; EconLit (EBSCOhost); PubMed; APA PsycINFO (Ovid); SCOPUS (Elsevier): Web of Science Core Collection (Clarivate) (Arts and Humanities Citation Index, Social Sciences Citation Index, Science Citation Index, Expanded Emerging Sources Citation Index, and Conference Proceedings Citation Index)

Trial registries: We will search the following trial registries: Clinicaltrials.gov and the International Clinical Trials Registry Platform (WHO; https://who.int/ictrp/network/en/). We will also search the Centers for Disease Control and Prevention website.

Website and online sources: Our search for unpublished studies will include the following relevant websites: World Bank, OECD, Global Partnership for Financial Inclusion, and Alliance for Financial Inclusion.

The reference lists from included studies and related systematic/scoping reviews will be reviewed for potential studies. We will also conduct forward citation searching using Google Scholar to search for studies citing included studies.

Authors of included studies will be contacted in an attempt to obtain unpublished studies, studies in process, and published studies missed in the database search.

#### 3.6.1 Search terms

We will use combinations of terms related to the intervention and study design to search the electronic databases. Database‐specific strategies will be explored for each database in consultation with a librarian at Saint Louis University, including the use of truncation and data‐base specific limiters and thesauri will be consulted to employ more precise search strategies within each database. We will use any available database filter to filter out nonhuman studies. See Appendix A for a sample search in the PubMed database. Below are examples of the types of terms we anticipate using:

a. Related to financial:
  ((((“FInancial stress” [Mesh:NoExp]) OR “financial toxicity” OR “financial stress” OR “financial strain” OR “financial capability” OR “financial services” OR “economic stress” OR “economic services”) OR
b. Related to interventions:
  (“financial coaching” OR “financial counseling” OR “financial mentoring” OR “financial education” OR “financial services” OR “benefit screen” OR “tax filing assistance” OR “VITA site” OR “VITA” OR “Connection to benefit” OR “free tax clinic” OR “earned income tax credit” OR “EITC” OR “child tax credit” OR “CTC” OR “child savings account*” OR “child development accounts” OR “credit counseling” OR “credit counsel*” OR “employment assistance” OR “FAFSA form assistance” OR “pre‐K enrollment assistance” OR medical bill arbitration” OR “tax‐time saving intervention” OR “matched savings accounts” OR “job assistance” OR “resume building” OR “savings class”) OR
c. Related to outcomes:
  (“reduce debt” OR “lower debt” OR “build credit” OR “increase credit” OR “build assets” OR “generate wealth” OR “grow income”) AND
d. Health
  (“health clinic” OR “primary clinic” OR “primary care clinic” OR “health system” OR “clinic‐based” OR “hospital” OR “clinical” OR “federally‐qualified health center” OR “pediatric” OR “pediatric medical home” OR “medical home‐embedded” OR “clinical‐community partnership” OR
e. Specialized term
  (“Medical‐financial partnership” OR “clinic based financial services” OR “antipoverty medicine”) AND
f. Report type
  (evaluation OR intervention OR treatment OR outcome OR program OR trial OR experiment OR “control group” OR “controlled trial” OR “quasi‐experiment” OR random* OR empirical OR research).

#### 3.6.2 Selection of studies

One reviewer will conduct the initial search in all sources and will save the search results in an electronic format (Endnotes) and remove duplicates. At this stage, the reviewer will upload the results into Rayyon to examine titles and abstracts and will discard results that are obviously ineligible (nonempirical report, book review, editorial, not human subjects, etc.). For those that are not obviously ineligible, the reviewer will retrieve the reports, save them in an electronic file, and document the bibliographic information, source, and date retrieved in a database. Two reviewers will then independently screen each of the reports for eligibility using a screening instrument (i.e., Rayyon), compare agreement between coders and identify any discrepancies. See Supporting Information: Appendix B for the screening tool that we plan to use. The review team will meet to discuss discrepancies and will resolve all discrepancies through consensus.

#### 3.6.3 Description of methods used in primary research

Studies examining effects of these interventions primarily use group design studies to compare differences between groups at the end of the intervention and, in some cases, additional follow up time points. For example, Schickedanz et al. (2023) used a RCT comparing clinic‐based financial coaching offered to the treatment group to usual care among low‐income parent‐infant dyads attending pediatric preventive care visits. They studied the outcomes of employment, savings, and public benefits enrollment through the 6‐month well‐child visit.

#### 3.6.4 Criteria for determination of independent findings

We are interested in two primary outcome constructs: outcomes related to finances and health. All financial outcomes will be included, which may include financial knowledge and attitude, tax filing status, tax refund receipt and amount, debt, savings, and credit scores. In addition, all health outcomes and perceptions about health will be included. These outcomes can include missed visits, immunization schedule adherence, and other health indicators. While we do not know what medical and financial outcomes we will locate, we plan to separate them into domains when possible. For example, we may be able to separate the medical outcomes into “Medical Behavior” (e.g., medical visit adherence, immunization schedule adherence) and “Medical Outcome” (e.g., lower stress, fewer sick child visits). Similarly, we may be able to separate the financial and economic outcomes into the domains of “Financial Behavior” (e.g., saving, filed taxes, purchased an asset, opened a Child Development Account, applied for a job) and “Financial Outcome” (e.g., saving amount, tax return amount, new job). Depending on the outcomes located, we may also be able to create a financial domain of “Financial Knowledge and Attitude” (e.g., results of financial knowledge assessment).

We anticipate that some included studies may use multiple measures for each outcome, multiple reports of the same outcome measure, multiple follow‐up points, more than one intervention, and possibly more than one counterfactual condition. These circumstances create statistical dependencies that violate assumptions of standard meta‐analytic methods. To ensure independence of study‐level effect sizes, we will include only one effect size estimate from each independent sample for each outcome construct in each meta‐analysis.

For cases in which a study uses multiple measures (i.e., observation and a standardized instrument) of the same construct, we will code data for each measure and create a study‐level average across measures. In cases of multiple reports on the same outcome (i.e., parent and child report), we will code data for each report and conduct separate meta‐analyses (i.e., similar types of reporters will be pooled). In cases where multiple points of follow‐up are provided, we will code follow‐up points to conduct a separate analysis for effect sizes comparing studies with similar points of follow‐up. In the case of multiple counterfactual conditions, we will select the comparison condition that is most similar to those in the other included studies. For studies in which multiple interventions are tested, we will select the intervention that meets the inclusion criteria for this review. If there is more than one intervention in the study that meets the criteria for this review, we will combine the intervention groups.

## 4 DETAILS OF STUDY CODING CATEGORIES

### 4.1 Data extraction and management

For all studies that pass the eligibility screening process described earlier, two reviewers will independently code all eligible studies using a structured data extraction form (See Supporting Information: Appendix C). Multiple reports on individual studies will be collated. The data extraction from will include items related to bibliographic information and source descriptors: methods and procedures; context, intervention characteristics; sample characteristics; and outcome data needed to calculate effect sizes.

Coders will pilot test the code form together using diverse types of studies and will discuss any items that are unclear and ensure mutual understanding of all items. Following pilot testing of the form, two coders will independently code 100% of the included studies. Coders will compare coding and will identify and discuss discrepancies, which will be resolved through consensus. If consensus cannot be reached between the two coders, a third member of the review team will be consulted to resolve the discrepancy. Initial discrepancies will be recorded and inter‐rater reliability will be reported. In addition to comparing codes between coders, we will also compare the magnitude and direction of effects as presented in the review with how data is presented in the primary study to check for agreement.

### 4.2 Assessment of risk of bias in included studies

Two review authors will independently assess the risk of bias in all included studies using the Cochrane Collaboration's risk of bias tool (see Supporting Information: Appendix D) (Higgins et al., 2011). We will assess risk of bias of each of the six following domains: allocation, blinding, complete outcome data, selective reporting, and other potential sources of bias (i.e., researcher allegiance, funding source). Each study will be coded as “low” “high” or “unclear” risk of bias on each of the domains. Following independent coding by two authors, coders will meet to identify any discrepancies, and all discrepancies will be resolved through consensus. If consensus cannot be reached between the two reviewers, a third member of the review team will be consulted.

Risk of bias in each domain will be reported within and across studies in the results section using narrative and graphs. We anticipate that most studies included in this review will be at high risk of bias in terms of location and blinding; thus, we do not plan to restrict analyses based on the risk of bias in any domain. We plan to present all included studies, provide the evidence and source used for the judgment, and provide a narrative discussion of the risk of bias to include discussion of the potential limitations of the review as well as implications of bias in the interpretation of the results in the Discussion section of the review.

### 4.3 Statistical procedures and conventions

We will conduct descriptive analyses on variables of interest from all included studies to provide information regarding:

- Study participants (e.g., subgroups, gender, race/ethnicity, income level, age; parent status).
- Setting where studies are situated (e.g., pediatric health clinics, tertiary settings)
- Funding mechanisms.
- Relevant intervention characteristics (e.g., type/duration of financial services, way in which the integration of services is characterized).
- Risk of bias across RCT and QED studies on each domain.

### 4.4 Measurement of treatment effect and unit of analysis

Following descriptive analysis, we will estimate the effect sizes for each outcome of interest in each included study. For the RCT and QED studies, we will calculate the magnitude of effect using the standardized mean difference effect size with Hedges’ *g* correction for continuous outcomes and odds ratios for outcomes presented as dichotomous variables. We anticipate that outcomes within a category will be measured using similar metrics and therefore, effect sizes within each category will only use either Hedges’ *g* or odds ratio metrics in the analysis. If, however, outcomes within one of the outcome categories are measured in different metrics, we will convert them to Hedges’ *g*. When studies use nonstandard designs (e.g., clustering) we will follow procedures per the What Works Clearinghouse Procedures and Standards Handbook version 3.0 (Institute of Education Sciences, 2014). All effect sizes will be coded so that a positive effect size is indicative of the treatment group positively outperforming the comparison group on the outcome. If primary studies do not report sufficient data to calculate an effect size, we will contact the study authors and request the data. If we are unable to obtain the data, we will include the study in the review, but it will not be included in the meta‐analysis. We interpret a statistically nonsignificant *p* value (e.g., larger than 0.05) as a finding of uncertainty unless confidence intervals are sufficiently narrow to rule out an important magnitude of the effect.

### 4.5 Data synthesis

Following the estimation of individual study‐level effects, we will conduct separate meta‐analyses to pool studies for each outcome construct. A weighted mean effect will be calculated by weighing each study‐level effect size by the inverse of its variance. Random effects statistical models will be used throughout unless a compelling case arises for fixed effect analysis. RCT studies will be pooled separately from QED studies; however, if RCT and QED studies are found to be homogenous, studies will be pooled to allow for greater statistical power. If we have multiple effect sizes per study (e.g., multiple measures of the same outcome construct or multiple treatment conditions against the same control condition), we will conduct meta‐analysis in R using the robumeta package (Fisher et al., 2017) to conduct robust variance estimation (RVE) with small‐sample corrections. RVE accounts for the nonindependent sampling errors due to inclusion of multiple effect sizes from the same study.

### 4.6 Assessment of heterogeneity

Following the estimation of summary effects, we will conduct a test of homogeneity (*Q* test) to compare the observed variance to what would be expected from sampling error. The *I* ^2^ statistic will also be used to describe the percentage of total variation across studies due to heterogeneity rather than chance. We will also construct a forest plot displaying study‐level mean effect sizes and 95% confidence intervals for the included studies to provide opportunity for visual analysis of the precision of the estimated effect sizes, detection of studies with extreme effects, and information regarding the heterogeneity of studies. Publication bias will be assessed using funnel plots.

Provided that there are a sufficient number of studies, we will conduct moderator analysis to examine whether characteristics of the study methods and interventions may be associated with effect size. The approach to moderator analysis will be dependent upon the available data. If there are sufficient studies available, meta‐regression will be used for continuous outcomes and the analog to the Analysis of Variance will be used for categorical variables. Moderating variables of interest include study design (RCT and QED), publication status (published or unpublished), dosage and duration of intervention (continuous variable), age (children or adults), and type of intervention (financial coaching, tax filing assistance, or other). We anticipate that most of these studies will be conducted with low income groups, and will include a mix of racial/ethnic groups, thus we do not anticipate being able to conduct moderator analysis related to SES/income or race of participants, but if there are studies that differentiate between different income groups, we plan to conduct moderator or subgroup analyses on household income and race.

### 4.7 Sensitivity analysis

Sensitivity analysis will be conducted to examine the potential effects of outliers.

*Summary of findings and assessment of the certainty of the evidence*

We do not plan to include a summary of findings and assessment of the certainty of the evidence.

## POTENTIAL CONFLICT OF INTEREST STATEMENT

We have no potential conflicts of interest. No authors have been involved in the development of related interventions, primary research on medical‐financial partnerships, or prior published review on this topic.

## PRELIMINARY TIMEFRAME

Date you plan to submit a draft review: May 15, 2024.

## PLANS FOR UPDATING THE REVIEW

The authors agree to update the review if sufficient new studies and adequate funding to undertake this venture becomes available.

## Supporting information

Supporting information.
