# Supplementary material for: PROTOCOL: Medical‐financial partnerships for improving financial and health outcomes for lower‐income Americans: A systematic review
Source: Campbell Syst Rev. 2023 Oct 9;19(4):e1364. doi: 10.1002/cl2.1364 (PMC10561025; doi:10.1002/cl2.1364)
Supplement: Supplementary file 1 — Supporting information. [file CL2-19-e1364-s001.docx]

**APPENDIX A**

Sample Search

PubMed - Advanced Search

Field: All Fields

Line-by-line search:

((((“Financial Stress”[Mesh:NoExp]) OR (“financial toxicity” OR “financial stress” OR “financial strain” OR “financial capability” OR “financial services” OR “economic stress” OR “economic services”)) OR (“financial coaching” OR “financial counseling” OR “financial mentoring” OR “financial education” OR “financial services” OR “benefit screen” OR “tax filing assistance” OR “VITA site” OR “VITA” OR “connection to benefit” OR “free tax clinic” OR “earned income tax credit” OR “EITC” OR “child tax credit” OR “CTC” OR “child savings account” OR “child development accounts” OR “credit counseling” OR “credit counsel” OR “employment assistance” OR “FAFSA form assistance” OR “pre-K enrollment assistance” OR “medical bill arbitration” OR “tax-time saving intervention” OR “matched savings accounts” OR “job assistance” OR “resume building” OR “savings class”))

OR

(“reduce debt” OR “lower debt” OR “build credit” OR “increase credit” OR “build assets” OR “generate wealth” OR “grow income”)))

AND

((“health clinic” OR “primary clinic” OR “primary care clinic” OR “health system” OR “clinic-based” OR “hospital” OR “clinical” OR “federally-qualified health center” OR “pediatric” OR “pediatric medical home” OR “medical home-embedded” OR “clinical-community partnership”) OR (“Medical-financial partnership” OR “clinic based financial services” OR “antipoverty medicine”))

AND

((evaluation OR intervention OR treatment OR outcome OR program OR trial OR experiment OR “control group” OR “controlled trial” OR “quasi-experiment” OR random* OR empirical OR research)

MeSH term used: “Financial stress”

Search in: All Fields

Time period: 1967-present

Filers:

English language

Human subjects

Report type: Address, Case Reports, Classical Article, Clinical Conference, Clinical Study, Clinical Trial, Clinical Trial Protocol, Clinical Trial, Phase I, Clinical Trial, Phase II, Clinical Trial, Phase III, Clinical Trial, Phase IV, Comparative Study, Congress, Consensus Development Conference, Consensus Development Conference, NIH, Controlled Clinical Trial, Corrected and Republished Article, Duplicate Publication, Electronic Supplementary Materials, English Abstract, Evaluation Study, Government Publication, Guideline, Historical Article, Interview, Introductory Journal Article, Lecture, Multicenter Study, Observational Study, Overall, Periodical Index, Pragmatic Clinical Trial, Preprint, Published Erratum, Randomized Controlled Trial, Research Support, American Recovery and Reinvestment Act, Research Support, N.I.H., Extramural, Research Support, N.I.H., Intramural, Research Support, Non-U.S. Gov’t, Research Support, U.S. Gov’t, Non-P.H.S., Research Support, U.S. Gov’t, P.H.S., Research Support, U.S. Gov’t, Retracted Publication, Retraction of Publication, Technical Report, Twin Study, Validation Study

Results: 67,003 records

**APPENDIX B**

Screening Form

**Is this study a**

- RCT
- QED with parallel cohort
- None of the above – IF CHECKED THEN STOP

**Was this study conducted in the United States?**

- No – STOP
- Yes
- Unsure

**Does this study involve an intervention that provides financial services on-site within a healthcare setting or via tele-consultation with a financial service provider who is providing services within a service delivery model of a healthcare provider?**

- No – no financial services provides - STOP
- No - not in the healthcare setting - STOP
- Yes
- Unsure

**Does this study measure an outcome related to finances? No – STOP**

- Yes
- Unsure

**Is this study eligible for the review?**

- No – Reason _____________
- Yes
- Need more information to make a decision

**APPENDIX C**

Data Extraction Form

**Report type**

1. Journal article
2. Book/book chapter
3. Government report (local, state, federal)
4. Conference proceedings
5. Thesis or dissertation
6. Unpublished report (non-government, technical report) and other
7. Research brief

**Intervention healthcare setting**

1. Pediatric clinic
2. Community healthcare clinic
3. Hospital
4. Ambulatory sites affiliated with a hospital
5. Primary health clinic
6. Older adult setting

**Patients/participants are**

1. Children and youth
2. Adults
3. Both

**Financial service provided**

1. Financial coaching
2. Financial literacy/education
3. Financial counseling
4. Credit/debt counseling
5. Free tax preparation
6. Matched college savings program
7. Employment services
8. Job training
9. One-on-one case management
10. Public benefits screening
11. Assistance in applying or obtaining public benefits
12. Budgeting assistance
13. Expense reduction
14. Savings promotion
15. FAFSA form assistance

**Other services provided**

1. Medical bill arbitration
2. Pre-K enrollment assistance
3. Housing assistance
4. Food insecurity assistance
5. Diaper distribution
6. Other: (fill in)

**Method of assignment to condition**

1. Random, simple
2. Random, after matching, stratification, blocking, etc.
3. Quasi-random assigned by some naturally occurring process
4. QED with parallel cohorts
5. Not specified/not enough information to determine

**Results of statistical comparisons of pretest differences on outcomes**

1. No statistical comparisons made
2. No statistically significant differences
3. Statistically significant differences found

**Results of statistical comparisons of pretest differences on demographics (race, ethnicity, income)**

1. No statistical comparisons made
2. No statistically significant differences
3. Statistically significant differences found

**Unit of assignment to conditions**

1. Individual participant
2. Group/cluster: specify:
3. Other
4. Not enough information to determine

**Mean age of participants**

Not specified

**Who provided the financial services?**

1. Nonprofit staff
2. For-profit company staff
3. Government/public staff
4. Researchers
5. Other
6. Not specified

**MFP funding mechanism**

1. Grants/philanthropy
2. hospital/clinic
3. Government
4. Loans/interest

**Patients are**

1. Primarily non-English speaking
2. Primary English speaking

**Predominant race/ethnicity**

1. African American
2. Asian
3. White, non-Hispanic
4. Hispanic
5. Not specified

**Sex**

1. Female %
2. Not specified

**Income**

1. Low Income
2. Low and moderate income
3. All income levels
4. Not specified

**If groups were non-equivalent at baseline, were statistical controls used?**

1. Yes
2. No
3. Were equivalent
4. Not enough information to determine

**If matching was used, how were groups matched?**

1. Matched on pretest measure
2. Matched on demographics
3. Matched on both of the above
4. Propensity Score Matching
5. Other matching technique
6. Not enough information to determine
7. Were not matched

**Role of the evaluator/author/research team or staff in the program**

1. Researcher independent of treatment – research only
2. Researcher not independent of treatment
3. Not specified

**Treatment format**

1. Individual (one-on-one) – customized treatment of some kind
2. Group – same tx for all subjects in tx group(s)
3. Individual and group mix of standard and customized tx
4. Other
5. Not specified

**Length of treatment**

1. Specified:
2. Not specified

**Frequency of contact**

1. Once
2. Periodically/irregular
3. Per week:
4. Per month:
5. As patient deems needed:
6. Not specified

**Total number of sessions**

1. Specified:
2. Not specified

**Is this intervention manualized?**

1. Yes
2. No
3. Not specified

**Did the study measure fidelity?**

1. Yes
2. No

**How was fidelity assessed?**

1. Not measured
2. Researcher observations
3. Interviews of participants
4. Surveys of participants
5. Participant logs
6. Administrative records
7. Checklists
8. Other
9. Not specified

**Did the treatment group have high attrition (for RCT/QED > 20%)?**

1. Yes
2. No
3. Not enough information to calculate

**Did the control group have high attrition (for RCT/QED > 20%)?**

1. Yes
2. No
3. Not enough information to calculate

**What did the control/comparison group receive?**

1. Nothing or wait list
2. Treatment as usual: specify
3. Specified treatment: specify
4. Other

**Timing of measurement of outcomes**

1. Tx baseline mean
2. Tx baseline SD
3. Tx baseline N
4. Tx post mean
5. Tx post SD
6. Tx post n
7. Control group baseline mean
8. Control group SD
9. Control group n
10. Values for t, F, other

Tx analytic sample size

Control group analytic sample size

**APPENDIX D**

Risk of Bias Tool

(Higgens et al., 2011)

| Bias domain | Source of bias | Support for judgment | Review author’s assessment (rate as low, unclear or high risk of bias) |
| --- | --- | --- | --- |
| Selection Bias | Random sequence generation | Describe the method used to generate the allocation sequence in sufficient detail to allow an assessment of whether it should produce comparable groups | Selection bias (biased allocation to interventions) due to inadequate generation of a randomised sequence |
|  | Allocation concealment | Describe the method used to conceal the allocation sequence in sufficient detail to determine whether intervention allocations could have been foreseen before or during enrolment | Selection bias (biased allocation to interventions) due to inadequate concealment of allocations before assignment |
| Performance bias | Blinding of participants and personnel | Describe all measures used, if any, to blind trial participants and researchers from knowledge of which intervention a participant received. Provide any information relating to whether the intended blinding was effective | Performance bias due to knowledge of the allocated interventions by participants and personnel during the study |
| Detection bias | Blinding of outcome assessment | Describe all measures used, if any, to blind outcome assessment from knowledge of which intervention a participant received. Provide any information relating to whether the intended blinding was effective | Detection bias due to knowledge of the allocated interventions by outcome assessment |
| Attrition bias | Incomplete outcome data | Describe the completeness of outcome data for each main outcome, including attrition and exclusions from the analysis. State whether attrition and exclusions were reported, the numbers in each intervention group (compared with total randomised participants), reasons for attrition or exclusions where reported, and any reinclusions in analyses for the review | Attrition bias due to amount, nature, or handling of incomplete outcome data |
| Reporting bias | Selective reporting | State how selective outcome reporting was examined and what was found | Reporting bias due to selective outcome reporting |
| Other bias | Anything else | State any important concerns about bias not covered in the other domains in the tool | Bias due to problems not covered elsewhere |
